# Tigecycline treatment in a liver transplant infant with carbapenem-resistant *Escherichia coli* infection: Case report

**DOI:** 10.1097/MD.0000000000017339

**Published:** 2019-09-27

**Authors:** Mei Yang, Hengmiao Gao, Xiaoling Wang, Suyun Qian

**Affiliations:** a Departments of Pharmacy; b PICU, Beijing Children's Hospital, Capital Medical University, National Center for Children's Health, Beijing, China.

**Keywords:** carbapenem-resistant, infant, liver transplantation, tigecycline

## Abstract

**Introduction:**

During the past decade, the rate of carbapenem resistance among Enterobacteriaceae, mostly in *Escherichia coli* and *Klebsiella pneumoniae*, has significantly increased worldwide. It is a great challenge for the choice of drug treatment especially in children.

Tigecycline is the first drug in the glycylcycline class of antibiotics. For children, the China Food and Drug Administration and US Food and Drug Administration postulated that tigecycline is not recommended. It must be used only as salvage therapy for life-threatening infections in critically ill children who have no alternative treatment options.

**Patient Concerns:**

A male pediatric case of 4.5 months was blood stream infection after liver transplantation. The blood cultures obtained grew Gram-negative rods, which reportedly grew a strain of extended-spectrum β-lactamase and carbapenemases-producing *Escherichia coli* within 10 hours. All bacterial isolates were found to be resistant to all antimicrobial agents except aminoglycosides and tigecycline.

**Diagnoses:**

Complicated intra-abdominal infection, central line-associated blood stream infection.

**Interventions:**

The blood stream infection with carbapenem-resistant *Escherichia coli* after liver transplantation was cured by tigecycline.

**Outcomes:**

The patient's condition continued to improve, then transferred to general ward.

**Conclusion:**

The following report, to our knowledge, is the youngest liver transplantation patient who used tigecycline treatment around the world. It provides reference and experience for the use of tigecycline in infants with severe infections.

## 1 Introduction

During the past decade, the rate of carbapenem resistance among Enterobacteriaceae, mostly in *Escherichia coli* and *Klebsiella pneumoniae*, has significantly increased worldwide. Carbapenem resistance can be due to various β-lactamases, but it has been associated frequently with carbapenemases (CPE).

World Health Organization published the global priority list of antibiotic-resistant bacteria to guide research, discovery, and development of new antibiotics on February 27, 2017.^[1]^ In the article, the experts agreed on grouping the pathogens according to the species and the type of resistance and then stratifying the results in 3 priority tiers: critical, high, and medium, and Enterobacteriaceae include *Klebsiella pneumonia*, *Escherichia coli*, *Enterobacter* spp, *Serratia* spp, *Proteus* spp, *Providencia* spp, and *Morganella* spp are carbapenem-resistant or 3rd generation cephalosporin-resistant is critical level.^[1]^

Tigecycline, a derivative of minocycline, is the first drug in the glycylcycline class of antibiotics. It has been approved by the US Food and Drug Administration (FDA) in 2005 for complicated skin and skin structure infections (cSSSIs), complicated intra-abdominal infections, and community-acquired pneumonia (CAP) in patients aged >18 years.^[2]^ It has a broad spectrum of activity including multidrug-resistant/extensively drug-resistant(MDR/XDR) Gram-positive and Gram-negative bacteria, with the exception of *Pseudomonas* spp.^[3–5]^ In 2012, it has been on the market in China and approved population, indications, and dosage by China Food and Drug Administration (CFDA) were no difference from US FDA.

We report a 4.5 months male pediatric case of blood stream infection with carbapenem-resistant Escherichia coli after liver transplantation, cured by tigecycline. The following report, to our knowledge, is the youngest liver transplantation patient who used tigecycline treatment around the world.

## 2 Case report

The boy was born on February 16th, 2017, diagnosed with congenital biliary atresia when he was 45 days’ old, received cadaveric liver transplantation from an unrelated donor on July 2nd, 2017 in Beijing Children's Hospital. Before surgery, he received antibiotic prophylaxis with cefoperazone sulbactam (50 mg/kg, 0.5 hours before surgery). After surgery on July 3rd, 2017, he was transferred to the pediatric intensive care unit (PICU) for close monitoring. A central venous catheter (CVC) was placed and, because he could not feed orally, he was placed on total parenteral nutrition. Empirical antibiotherapy was initiated with imipenem/cilastatin sodium (15 mg/kg, every 6 hours).

On day 6 in PICU, physical examination revealed a confused patient with a peak temperature of 39.4°C, a blood pressure of 80/40 mmHg, a pulse of 120 to 140 beats/min, and a respiratory rate of 50 to 70 breaths/min. Laboratory studies showed white blood cell (WBC) was18.33 × 10^9^ cells/L with 61.7% neutrophils, C-reactive protein (CRP) was 44 mg/L, procalcitonin (PCT) was 6.62 ng/mL. The celiac effusion and peripheral blood cultures obtained grew Gram-negative rods, which reportedly grew a strain of ESBL and CPE-producing *Escherichia coli* within 10 hours. All bacterial isolates were found to be resistant to all antimicrobial agents except aminoglycosides and tigecycline. The patient developed abdominal infection and bacteremia, so the imipenem/cilastatin sodium was discontinued; antibiotics were modified tigecycline (1.2 mg/kg q12 h) and tobramycin (2 mg/kg q8 h) according to the results of drug sensitivity. Removed the CVC, started enteral nutrition, then gradually transition to breastfeeding. Three days later, laboratory studies showed WBC was 48.95 × 10^9^ cells/L with 55% neutrophils, CRP was 74 mg/L, PCT was 0.27 ng/mL. Treatment was continued for day 14 in PICU, and laboratory studies showed WBC was 12.37 × 10^9^ cells/L with 40.9% neutrophils, CRP was 11 mg/L, PCT was 0.24 ng/mL. Multiple subsequent blood cultures remained negative. Physical examination revealed a confused patient with a peak temperature of 36.8°C, a blood pressure of 80/40 mmHg, a pulse of 120 to 130 beats/min, and a respiratory rate of 30 to 40 breaths/min. The patient's condition continued to improve, tobramycin was discontinued, and was transferred to general ward.

Tigecycline has been continued for 7 days in general ward; laboratory studies at that time revealed a WBC of 11.85 × 10^9^/L with 37.4% neutrophils, CRP of <8 mg/L, and PCT of <0.05 ng/mL, and then tigecycline was discontinued. Our patient recovered uneventfully and without any apparent adverse drug interactions. Twenty-two days after surgery on July 24th, 2017, the outcome in this pediatric patient was improved greatly and then discharged.

### 2.1 Ethics statement

Tigecycline is off-label use for infants in China. We used tigecycline and tobramycin as the salvage therapy in this case and according to the susceptibility results. Ethical approval was not necessary for the case report, but informed consent was obtained from the patient's parents for publication of this case report and accompanying images, and tigecycline treatment was approved by pharmacy and therapeutics committee of the hospital.

## 3 Discussion

In this report, we present an infant's case who received tigecycline as the salvage therapy for serious infections owing to XDR bacteria in liver transplantation. When CPE isolates evolve to XDR, it clearly becomes a life-threatening situation requiring extraordinary treatment. The combination of different antibiotics with synergistic mechanisms of action not only may be useful for the management of XDR Gram-negative infections but also can lessen the chance of resistance development. Based on the drug susceptibility analysis in this case, the combination of tigecycline and tobramycin was successful in eradicating the infection without the apparent development of additional drug resistance. For children, the CFDA postulated that tigecycline is not recommended especially in patients younger than 8 years,^[6]^ the same as US FDA, unless no alternative antibacterial drugs are available. The suggested doses are 1.2 mg/kg of tigecycline every 12 hours i.v. to a maximum dose of 50 mg every 12 hours for children aged 8 to 11 years and 50 mg every 12 hours for adolescents aged 12 to 17 years.^[2,7]^ The European Medicines Agency has approved tigecycline treatment for infectious diseases in children above 8 years of age; the suggested dose is 1.2 mg/kg every 12 hours i.v. to a maximum dose of 50 mg every 12 hours and duration of therapy is from 5 to14 days.^[8]^ Tigecycline has an extensive distribution and higher concentration in human tissue outside of the plasma volume,^[9]^ a high-dose regimen as used for blood stream infections in adult patients,^[10]^ and blood stream infections have been a successful treatment by tigecycline in children with the following details.^[11–15]^

Foresti et al^[11]^ described in 2 pediatric oncohematological patients, tigecycline locks therapy for intravascular catheter-related infection owing to *Klebsiella pneumoniae* carbapenemases (KPC)-producing *Klebsiella pneumoniae*. Because of its lipophilic nature, which enables it to penetrate the biofilm, reaching the bacteria embedded in it, and its stability at concentrations that confer bactericidal activity, tigecycline is considered a suitable lock therapy agent. It can be used for cases of MDR Gram-negative strain where removing the CVC entails significant treatment burdens.

Dinleyici et al^[12]^ described tigecycline treatment of MDR *Corynebacterium jeikeium* infection in a 6-year-old female with relapsing and refractory acute lymphoblastic leukemia. They used tigecycline 1 mg/kg i.v. every 12 hours without the removal of the CVC; the patient recovered clinically.

Zeng et al^[13]^ described a 1-year old liver transplant case with blood stream infection of *Acinetobacter baumannii* successfully treated with tigecycline. Tigecycline (1 mg/kg q12 hours) serum concentrations were monitored by LC/MS/MS and its concentrations ranged from 111.5 to 159.0 μg/L. There was no dose reduction or discontinuation of tigecycline owing to adverse drug reaction, so the dosage regimen was safe and effective.

Zhu et al^[14]^ retrospectively analyzed 24 patients with tigecycline treated in a Chinese tertiary centre. Tigecycline was administered at a loading dose of 1.5 or 2.0 mg/kg and 1.0 mg/kg every 12 hours after that. The average duration of treatment was 11.6 ± 5.8 days. The clinical response and microbiological eradication rate were 37.5% and 29.2%, respectively.

Iosifidis et al^[15]^ reviewed 13 children with a median age of 8 years (2.5 months–14 years) who received tigecycline for ≥2 days as treatment for healthcare-associated infections. A loading dose (range, 1.8–6.5 mg/kg) was given in all except 2 cases. Maintenance dose was given at 1 to 3.2 mg/kg q12 hours. It seemed to be well tolerated in a series of mainly critically ill pediatric patients. This case series collects the largest number of pediatric patients treated with tigecycline.

Du et al^[16]^ presented a case with tigecycline on a 14-year-old child who was suffering from infection of intraperitoneal abscess caused by KPC-producing *Escherichia coli* with extreme drug-resistant profile. The patient received tigecycline (first dose of 100 mg, then 50 mg q12 hours, according to1 mg/kg body weight) for 6 days. During the therapy, the patient developed vomiting, which was thought to be probably related to tigecycline.

Mastrolia et al^[17]^ conducted a systematic review of the use of tigecycline in children. Sixty-two cases regarding the use of tigecycline in pediatric age were identified in the available literature. The mean age was 4.45 years (range 50 days–14 years) and 38.7% of patients were <3 years’ old. Tigecycline was used to treat cSSSI, CVC infections, CAP, urinarytract infections, peritonitis, biliary tract infections, meningitis, bacteremia, and sepsis. The reported dosage and treatment duration varied.

Pharmacokinetics (PK) data does not exist for infants and children younger than 8 years.^[18]^ Whether use loading dose or not in children is still unknown. In most pediatric cases, tigecycline was used at a dose of 1 to 1.2 mg/kg q12 hours. We also used 1.2 mg/kg every 12 hours without loading dose.

The penetration of tigecycline into the cerebrospinal fluid (CSF) is minimal, so the PK of tigecycline do not support its use to treat meningitis.^[19]^ Despite low CSF concentrations of tigecycline relative to the minimum inhibitory concentration, there are case reports in children and adults describing successful treatment of MDR central nervous system infections.^[20–24]^

According to the published literature, this may be the youngest liver transplant patient successfully treated with tigecycline. The risks and benefits of tigecycline regimen should be carefully weighted when making therapeutic decisions. Long-term adverse events, such as permanent discoloration of teeth, hearing impairment, should also be considered. However, the optimal dose of tigecycline, and whether to use a loading dose or not in children remains unknown.

## 4 Conclusions

In conclusion, tigecycline must be used only as salvage therapy for life-threatening infections in critically ill children who have no alternative treatment options. In this case, infections were controlled successfully, and it is efficient and safe of tigecycline in treating the pediatric patient. It provides reference and experience for the use of tigecycline in infants with severe infections.

Further studies are needed regarding the PK, efficacy, and safety of tigecycline in pediatric patients. The aim of future clinical trials should be the appropriate antibiotic dosage and duration of therapy for different infections and in different pediatric age groups especially younger than 8 years.

## Author contributions

**Data curation:** Hengmiao Gao.

**Resources:** Xiaoling Wang.

**Writing – original draft:** mei yang.

**Writing – review & editing:** Suyun Qian.
